# Activation of FGD5-AS1 Promotes Progression of Cervical Cancer through Regulating BST2 to Inhibit Macrophage M1 Polarization

**DOI:** 10.1155/2021/5857214

**Published:** 2021-10-15

**Authors:** Guokun Liu, Xuan Du, Li Xiao, Qing Zeng, QianLing Liu

**Affiliations:** ^1^Outpatient Department, Huai'an Second People's Hospital, The Affiliated Huai'an Hospital of Xuzhou Medical University, Huai'an, China; ^2^Department of Gynaecology and Obstetrics, The Central Hospital of Wuhan, Tongji Medical College, Huazhong University of Science and Technology, Wuhan, China; ^3^Department of Network Medicine, The Central Hospital of Wuhan, Tongji Medical College, Huazhong University of Science and Technology, Wuhan, China; ^4^Department of Clinical Laboratory, Hospital of Chengdu University of Traditional Chinese Medicine, Chengdu, China

## Abstract

Accumulating evidence has elucidated the biological function of lncRNAs in various tumors. FGD5 antisense RNA 1 (FGD5-AS1) is identified as a significant tumor regulator in malignancies. Up to now, the detailed function of FGD5-AS1 in cervical cancer and its underlying molecular mechanisms remain uninvestigated. Bone marrow stromal cell antigen 2 (BST2) can play critical roles in immune response, and the roles of BST2 in cervical cancer was explored currently. The level of FGD5-AS1 and BST2 was detected by qRT-PCR in cervical cancer cells. FGD5-AS1 and BST2 expression was significantly upregulated in cervical cancer cells. Then, the decrease of FGD5-AS1 greatly repressed cervical cancer cell growth in vitro. In addition, FGD5-AS1 silencing repressed BST2 expression and suppressed M2 macrophage polarization. Mechanistically, we confirmed that FGD5-AS1 sponged miR-129-5p to reduce its inhibition on BST2. Furthermore, lack of BST2 depressed cervical cancer cell growth, while inducing apoptosis. Loss of BST2 induced M1 macrophage polarization while blocking M2 macrophage polarization. For another, we demonstrated that FGD5-AS1-triggered M2 macrophage polarization was remarkably reversed by miR-129-5p via suppressing BST2. In conclusion, FGD5-AS1 induced M2 macrophage polarization via sponging miR-129-5p and modulating BST2, thus contributing to cervical cancer development. Our findings revealed FGD5-AS1/miR-129-5p/BST2 as a new potential target for cervical cancer.

## 1. Introduction

Cervical cancer is the fourth frequent cancer among females, which can account for 7.5% of female cancer deaths worldwide [1]. It has been reported that human papillomavirus (HPV), early sexual behavior, sexual relations with several partners, and smoking can contribute to cervical cancer [2, 3]. Up to now, radiotherapy, radical hysterectomy, and platinum-based chemotherapy are indicated as the standard primary treatment for cervical cancer [4]. Consequently, exploring the mechanism of cervical cancer and looking for suitable therapeutic targets might be helpful for treating cervical cancer.

Macrophages exhibit a central role in host defense [5]. In recent years, the importance of the immune microenvironment in cancers has gained much attention. Tumor-associated macrophages are an important component of the tumor microenvironment. Tumor-associated macrophages can differentiate into antitumor classical activation type (M1 type) or protumor alternative activation type (M2 type) [6]. Furthermore, an increased number of M2 tumor-associated macrophages have been highly correlated with tumor metastasis [7].

lncRNAs are transcripts with over 200 bp nucleotides with poor protein-coding potential [8, 9]. Aberrant ncRNAs are involved in many biological processes, including DNA methylation, histone modification, and transcription [10, 11]. lncRNAs can exert a critical role in various cancers [12]. Recently, abnormal lncRNAs are identified in cervical cancer development [13–15]. For example, lncRNA XLOC_006390 can induce cervical cancer through sponging miR-331-3p and miR-338-3p [16]. CASC11 contributes to cervical cancer via activating the Wnt pathway [17]. In addition, lncRNA SNHG7 induces cervical cancer cell growth [18].

FGD5-AS1 is expressed in multiple cancers. FGD5-AS1 can induce oral cancer growth via modulating MCL1 and sponging miR-153-3p [19]. FGD5-AS1 regulates gastric cancer via modulating miR-153-3p and CITED2 [20]. For another, knockdown of FGD5-AS1 decreases lung cancer viability, migration, and invasion through the modulation of miR-944 and MACC1 [21]. Nevertheless, the molecular mechanism of FGD5-AS1 in cervical cancer is unexplored.

BST2 is identified as CD317 [22]. It can play important roles in innate immune response [23]. In recent studies, BST2 is aberrantly expressed in cancers. For instance, BST2 promotes cell proliferation and migration through activating NF-*κ*B in gastric cancer [24]. Aberrant regulation of BST2 enhances breast cancer cell proliferation and apoptosis evasion [25]. However, its functional roles in cervical cancer require more investigation.

Taken these together, we found that FGD5-AS1 was upregulated in cervical cancer cells. Knockdown of FGD5-AS1 repressed the proliferation, migration, and invasion and increased apoptosis in vitro. Overexpressed, FGD5-AS1 promoted M2 tumor-associated macrophage polarization. Further, miR-129-5p was one of the targets of FGD5-AS1, and BST2 acted as a target for miR-129-5p. It was hypothesized that downregulation of FGD5-AS1 inhibited cervical cancer by inducing M2 tumor-associated macrophage polarization via targeting miR-129-5p and regulating BST2.

## 2. Methods and Materials

### 2.1. Cell Culture

HeLa, SiHa, C33A, CasKi, and H8 cells were obtained from Shanghai Institutes for Biological Sciences. Cells were incubated in DMEM containing 10% FBS and 50 *μ*g/mL penicillin and 50 *μ*g/mL streptomycin in an incubator at 37°C with 5% CO_2_. THP-1 were purchased from ATCC (Manassas, VA, USA). THP-1 cells were cultured in RPMI 1640 added with 10% FBS and 50 *μ*g/mL penicillin and 50 *μ*g/mL streptomycin.

### 2.2. Cell Transfection

pcDNA-FGD5-AS1 overexpression vector and the NC vector were constructed by GenePharma (Shanghai, China). FGD5-AS1/BST2 shRNA (shFGD5-AS1/BST2) or shRNA control (sh-NC), miR-129-5p mimics, inhibitors, or NCs were purchased from GenePharma (Shanghai, China). The Lipofectamine 3000 transfection reagent (Invitrogen, USA) was carried out based on the manufacturer's instruction.

### 2.3. Cell Proliferation Assay

Transfected SiHa cells in the same amount were plated in a 96-well plate, and cell proliferation was tested with CCK-8 (Dojindo, Tokyo, Japan) at 24 h and 48 h. A microplate reader (Bio-Rad, USA) was utilized to test the absorbance at 570 nm.

### 2.4. TUNEL Staining

After cells were transfected, cells were cultured for a whole night. After cells were fixed using 4% paraformaldehyde, cells were permeabilized in 0.25% Triton-X 100. Then, TUNEL assays were conducted. In brief, cells were treated in a terminal deoxynucleotidyl transferase reaction cocktail for 45 min. Then, incubation with the Click-iT reaction cocktail was followed.

### 2.5. Transwell Assay

To carry out the Transwell migration assay, cells were seeded in the upper chamber of each insert with the noncoated membrane. Lower chambers were added with 600 *μ*L medium added by 1% FBS. After 24 h, the cells on the lower surface were stained with 0.1% crystal violet. To do the invasion assay, matrigel chambers were carried out. Transfected cells were harvested in medium without serum. Then, the bottom chambers were incubated in 500 *μ*L DMEM added with 10% FBS. Afterwards, the invasive cells on the lower surface were stained with 0.1% crystal violet.

### 2.6. Real-Time PCR

Total RNA was extracted by the TRIzol® reagent. Next, extracted RNA was reverse transcribed into cDNA by the ReverTra Ace qPCR RT Kit (Toyobo, Japan). Quantitative PCR was performed using THUNDERBIRD SYBR®qPCR Mix and a LightCycler 480 Real-Time PCR system. Primer sequences are exhibited in Table 1. Gene expression was calculated according to 2^−*ΔΔ*CT^.

### 2.7. Western Blotting

In brief, cells were lysed using RIPA lysis buffer. 30 *μ*g proteins were exposed to 10% SDS-PAGE electrophoresis and then transferred to PVDF membranes. 5% nonfat milk was used to block the membranes for 1 h, and the membranes were incubated with primary antibodies overnight at 4°C. Antibodies against human BST2 and GAPDH were purchased from Cell Signaling Technology (Boston, MA, USA). The membranes were incubated with a secondary antibody for 1 h. Immobilon Western Chemiluminescent HRP substrate was used to visualize the protein bands.

### 2.8. Flow Cytometry

To evaluate the frequencies of CD206/CD163 and CD80/CD86, cells were stained with anti-CD80-APC, anti-CD86-PE, anti-CD206-FITC, or anti-CD163-APC (BD Biosciences, San Jose, California, USA). Staining with fluorophore-conjugated secondary antibodies was used for flow cytometry analysis.

### 2.9. Dual-Luciferase Reporter Assay

The predicted binding sequence of miR-129-5p in FGD5-AS1 (FGD5-AS1-WT) or BST2 3′UTR (BST2-WT) and their mutated sequence (FGD5-AS1-MUT and BST2-MUT) were separately cloned into pmirGLO vector. Mutagenesis of the predicted miR-129-5p seed sequence within the 3′UTR of FGD5-AS1 and BST-2 was performed by the GeneArt Site-Directed mutagenesis system (Invitrogen). To carry out the luciferase assay, SiHa cells were transfected with the above constructs and cotransfected with miR-129-5p NC or miR-129-5p mimics. 48 h later, luciferase activities were tested by the Dual-Glo® Luciferase Assay System (Promega, Madison, WI, USA).

### 2.10. RIP Assay

The RIP assay was carried out with the RIP™ RNA-Binding Protein Immunoprecipitation Kit from EMD Millipore. Briefly, SiHa cells transfected with miR-129-5p NC or inhibitor were collected. Then, cell lysate was incubated with RIP buffer containing magnetic beads conjugated with an anti-Ago2 antibody or IgG isotype control. Afterwards, RT-qPCR analysis was employed to evaluate the immunoprecipitated RNA.

### 2.11. Statistical Analysis

Statistical analysis was carried out using Student's *t*-test between two groups or one-way ANOVA followed by Tukey's post hoc test for multiple comparisons. *P* < 0.05 was considered to be statistically significant.

## 3. Results

### 3.1. FGD5-AS1 and BST2 Expression in Cervical Cancer Cells

**Figure 1:**
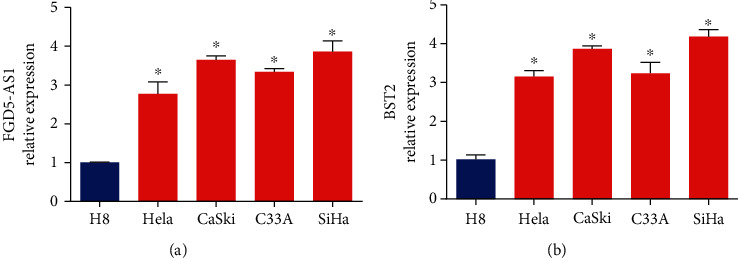
FGD5-AS1 and BST2 expression in cervical cancer cells. (a) RNA levels of FGD5-AS1 in HeLa, SiHa, C33A, CasKi, and H8 cells. (b) RNA levels of BST2 in HeLa, SiHa, C33A, CasKi, and H8 cells. ^∗∗∗^*P* < 0.001 and ^∗^*P* < 0.05. Three independent assays were carried out.

Firstly, quantitative RT-PCR was used to determine FGD5-AS1 and BST2 expression in cervical cancer cells (HeLa, SiHa, C33A, and CasKi cells) and H8 cells as shown in Figures 1(a) and 1(b). FGD5-AS1 and BST2 expression was obviously upregulated in cervical cancer cells (Figures 1(a) and 1(b)). Therefore, we speculated that FGD5-AS1 might promote cervical cancer development.

### 3.2. Effects of FGD5-AS1 on Cervical Cancer Cell Aggressiveness

**Figure 2:**
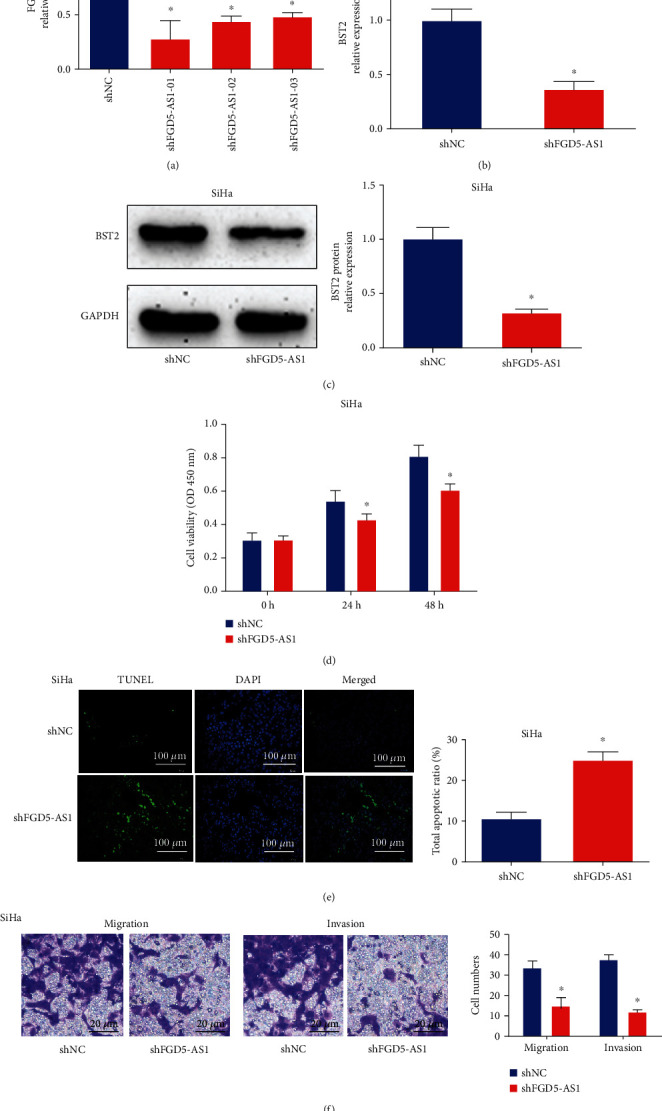
Effects of FGD5-AS1 on cervical cancer cell aggressiveness. (a) FGD5-AS1 expression in SiHa cells. FGD5-AS1 knockdown was generated in the SiHa cells by transfection of shFGD5-AS1. The transfection efficiency was validated by real-time PCR. (b, c) BST2 mRNA and protein expression in SiHa cells. (d) Cell viability by CCK8 assay. (e) Cell apoptosis by TUNEL assay. (f) Migration and invasion capacity using Transwell assay. ^∗^*P* < 0.05. shNC shRNA: negative control. Three independent assays were carried out.

To validate our hypothesis, we transfected shFGD5-AS1 into SiHa cells to generate FGD5-AS1 9 knockdown cervical cancer cells and carried out real-time PCR to verify the transfection efficiency. As exhibited in Figure 2(a), FGD5-AS1 shRNA-01 displayed the best knockdown effect on SiHa cells. In Figures 2(b) and 2(c), BST2 mRNA and protein expression was greatly reduced by the lack of FGD5-AS1. After knocking down FGD5-AS1 in cervical cancer cells, cell viability, cell apoptosis, migration capacity, and invasive capacity were assessed. In Figure 2(d), we found that silencing FGD5-AS1 repressed SiHa cell viability evidenced by the CCK assay. SiHa cell apoptosis was triggered by the downregulation of FGD5-AS1 as evaluated using the TUNEL assay in Figure 2(e). As demonstrated in Figure 2(f), SiHa migration and invasive capacity were significantly reduced by decreased FGD5-AS1. Thus, knocking down FGD5-AS1 inhibited the aggressiveness of cervical cancer cells.

### 3.3. Effects of FGD5-AS1 on Cervical Cancer Macrophage M2 Polarization

**Figure 3:**
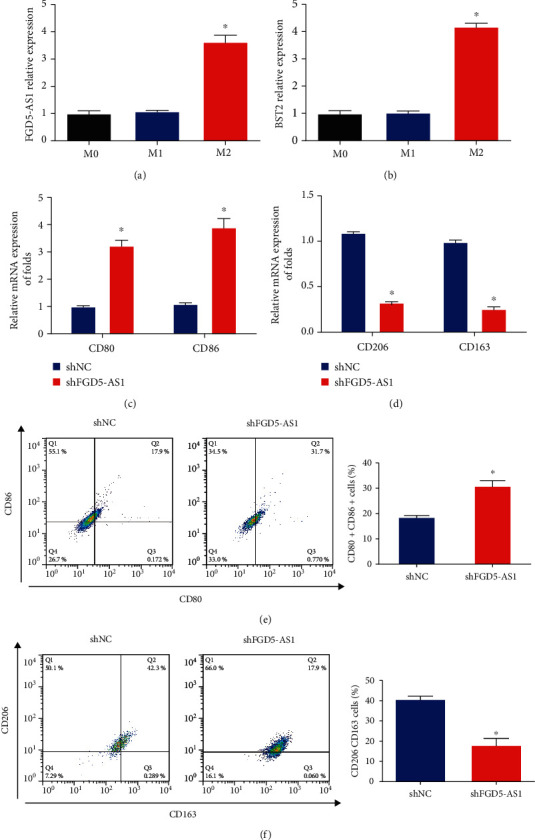
Effects of FGD5-AS1 on M2-like polarization of macrophages. (a) The expression of FGD5-AS1 was increased in M2 macrophages. (b) The expression of BST2 was upregulated in M2 macrophages. M0 cells were transfected with FGD5-AS1 shRNA. (c) Expression of M1 markers (CD80 and CD86). (d) Expression of M2 markers (CD206 and CD163). (e) Flow cytometry analysis of CD80 and CD86 in different transfectants of M0 cells. (f) Flow cytometry analysis of CD206 and CD163 in different transfectants of M0 cells. ^∗^*P* < 0.05. Three independent assays were carried out.

Furthermore, tumor-associated macrophages can exhibit an M2-like phenotype in the tumor microenvironment. In order to investigate whether FGD5-AS1 induced M2 polarization, we obtained unpolarized macrophages (M0), LPS/INF-*γ*-induced M1 macrophages, and IL-4/IL-13-induced M2 macrophages. Next, in Figures 3(a) and 3(b), lncRNA FGD5-AS1 and BST2 expression was increased in M2 macrophages compared to M1 macrophages. To block FGD5-AS1 expression, M0 cells were transfected with FGD5-AS1 shRNA. Then, polarization was induced in M0 cells, and the expression of M1 markers and M2 markers was evaluated. The results in Figure 3(c) indicated that M1 markers (CD80 and CD86) were significantly enhanced. In Figure 3(d), M2 markers (CD206 and CD163) were significantly reduced. Furthermore, flow cytometry analysis was performed, and we found that M1 markers (CD80 and CD86) were enhanced while M2 markers (CD206 and CD163) were downregulated by shRNA of FGD5-AS1 in Figures 3(e) and 3(f).

### 3.4. lncRNA FGD5-AS1 Sponged miR-129-5p to Modulate BST2

**Figure 4:**
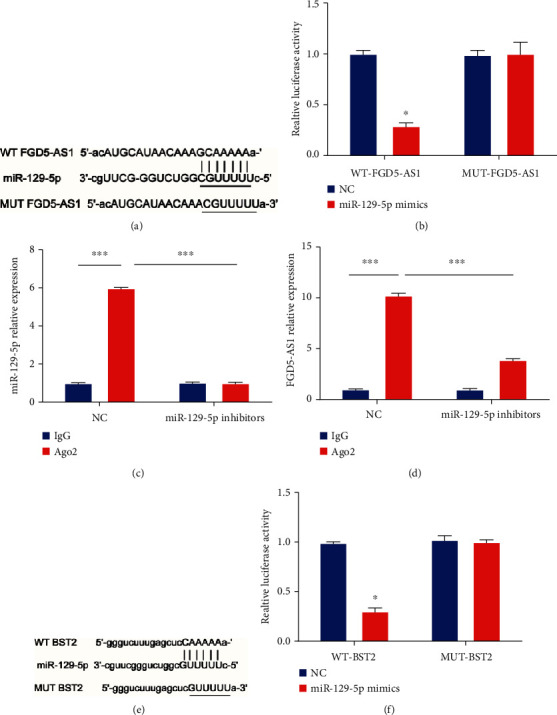
FGD5-AS1 sponged miR-129-5p to regulate BST2. (a) Binding site between FGD5-AS1 and miR-129-5p. (b) The luciferase activity in FGD5-AS1-WT and FGD5-AS1-MUT after transfection with miR-129-5p mimics or NC. (c, d) Enrichment of FGD5-AS1 and miR-129-5p in the anti-Ago2 or IgG immunoprecipitates in SiHa cells transfected with miR-129-5p NC or inhibitors. (e) The predicted binding site between BST2 and miR-129-5p. (f) The luciferase activity in BST2-WT and BST2-MUT after transfection with miR-129-5p mimics or NC. ^∗^*P* < 0.05. Three independent assays were carried out.

Then, it was predicted that FGD5-AS1 harbored one putative miR-129-5p binding site (Figure 4(a)). We confirmed whether miR-129-5p was a functional target of FGD5-AS1. Then, we observed that the luciferase activity was repressed by cotransfection of miR-129-5p mimics and FGD5-AS1-WT, suggesting that FGD5-AS1 is a direct target of miR-129-5p (Figure 4(b)). In Figures 4(c) and 4(d), both FGD5-AS1 and miR-129-5p were highly enriched in the anti-Ago2 pellet, which was reversed by miR-129-5p inhibitors. FGD5-AS1 directly sponged miR-129-5p. Moreover, miR-129-5p might directly target the 3′UTR of BST2 (Figure 4(e)); we observed that miR-129-5p mimics remarkably inhibited the luciferase activity with BST2-WT reporter plasmid (Figure 4(f)), which suggested that BST2 might be a direct target of miR-129-5p. It was manifested that FGD5-AS1 could sponge miR-129-5p to modulate BST2.

### 3.5. BST2 Promoted Cervical Cancer Progression via Inducing M2 Macrophage Polarization

**Figure 5:**
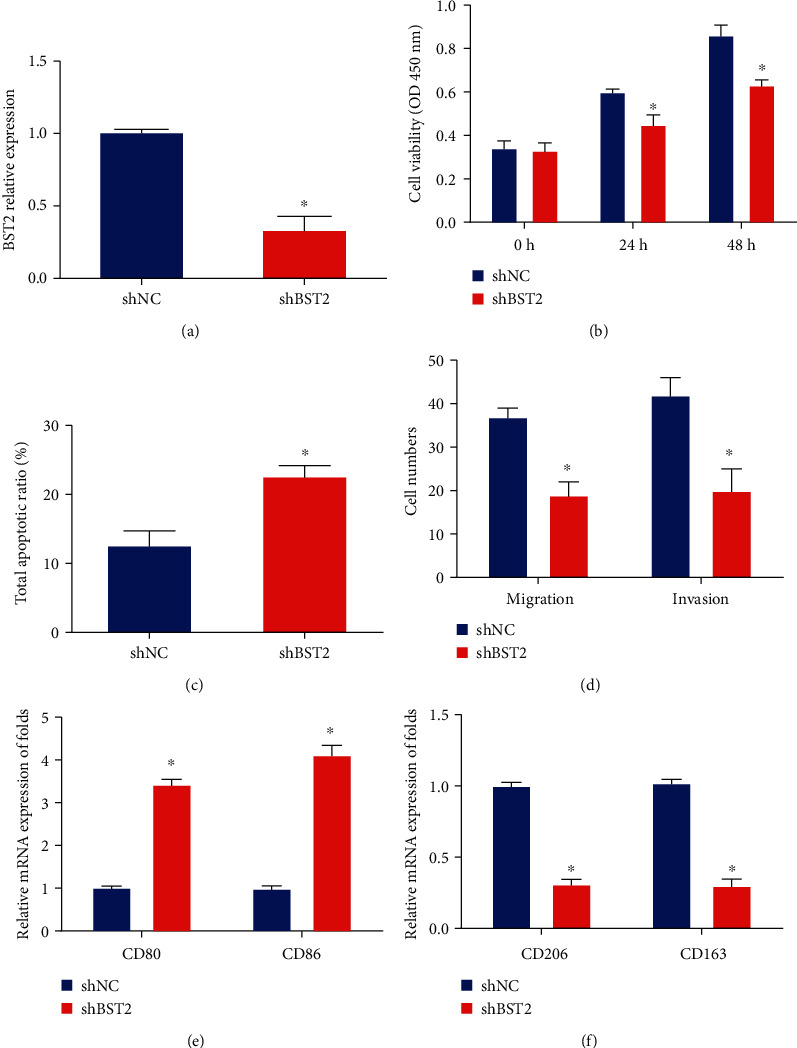
BST2 promoted cervical cancer progression via inducing M2 macrophage polarization. (a) mRNA levels of BST2 in SiHa cells transfected with BST2 shRNA. (b) Cell viability by CCK8 assay. (c) Cell apoptosis by TUNEL assay. (d) Migration and invasion capacity by Transwell assay. (e) mRNA expression of CD80 and CD86 in macrophages. (f) mRNA expression of CD206 and CD163 in M0 macrophages transfected with BST2 shRNA. ^∗^*P* < 0.05. Three independent assays were carried out.

In order to verify whether BST2 participate in cervical cancer progression via regulating M2 macrophage polarization in cervical cancer cells, SiHa cells were transfected with BST2 shRNA. As shown in Figure 5(a), BST2 was significantly reduced by BST2 shRNA in vitro. In Figures 5(b) and 5(c), loss of BST2 inhibited SiHa cell viability while SiHa cell apoptosis was induced after BST2 shRNA transfection. In Figure 5(d), cervical cancer cell migration and invasive capacity were suppressed by BST2 shRNA. In Figures 5(e) and 5(f), M0 cells were transfected with BST2 shRNA. Then, polarization was induced in M0 cells, and the expression of M1 markers and M2 markers was evaluated. It was indicated that M1 markers (CD80 and CD86) were upregulated while M2 markers (CD206 and CD163) were repressed by the silence of BST2.

### 3.6. miR-129-5p Mimics Reversed FGD5-AS1-Induced Upregulation of BST2 and M2 Macrophage Polarization

**Figure 6:**
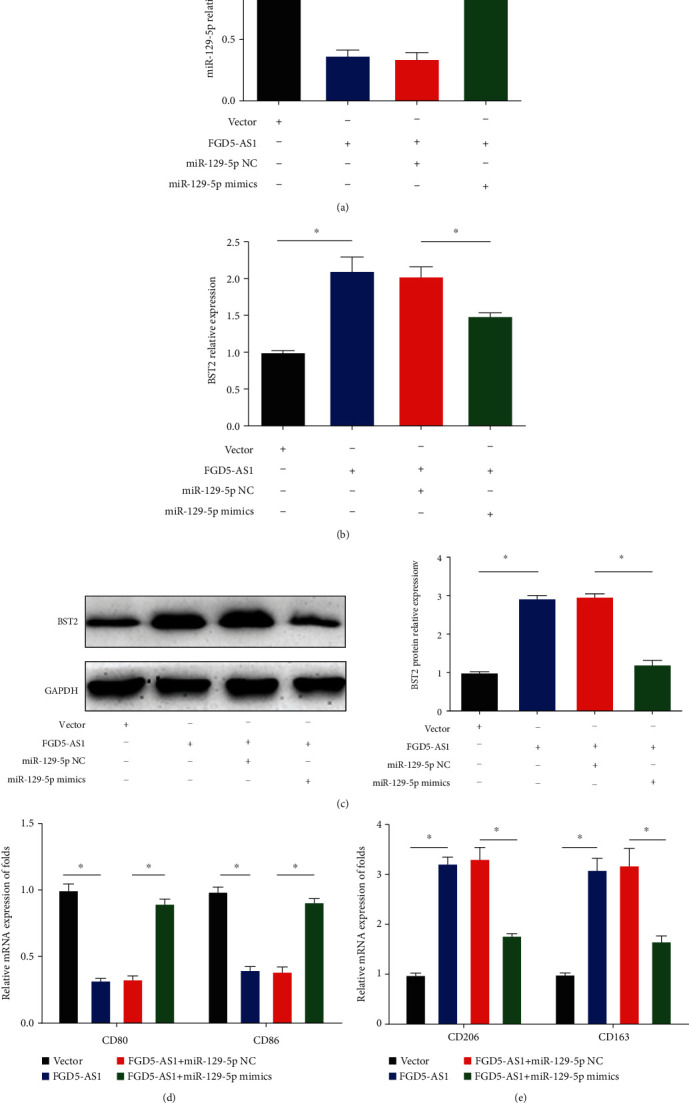
miR-129-5p mimics reversed FGD5-AS1-induced upregulation of BST2 and M2 macrophage polarization. (a) Levels of FGD5-AS1 in SiHa cells transfected with vector, FGD5-AS1, FGD5-AS1 plus miR-129-5p NC, or FGD5-AS1 plus miR-129-5p mimics. (b, c) The levels of BST2 in SiHa cells. (d) mRNA expression of CD80 and CD86 in macrophages. (e) mRNA expression of CD206 and CD163 in macrophages. ^∗^*P* < 0.05. Three independent assays were carried out.

SiHa cells were cotransfected with FGD5-AS1 overexpression plasmid and miR-129-5p mimics. miR-129-5p and BST2 expression levels were tested. Our data showed that overexpression of FGD5-AS1 obviously reduced miR-129-5p and induced BST2 expression. However, miR-129-5p mimics completely reversed the effects induced by FGD5-AS1 as shown in Figures 6(a)–6(c). Moreover, FGD5-AS1 activated M2 macrophage polarization. Then, we evaluated whether miR-129-5p mimics could reverse the effects of FGD5-AS1 on M2 macrophage polarization. miR-129-5p mimics enhanced the expression of M1 macrophage markers, while reducing M2 macrophage marker expression (Figures 6(d) and 6(e)). Overall, these indicated that FGD5-AS1 regulated BST2 and M2 macrophage polarization through sponging miR-129-5p.

## 4. Discussion

Recently, lncRNAs are reported to demonstrate tumor-inhibitory or tumor-promoting roles in cancers [26]. Then, many lncRNAs are shown to be associated with cervical cancer and serve as a promising biomarker for cervical cancer [27, 28]. Our present study clarified the biological significance of FGD5-AS1 in cervical cancer. In our research, we found that lncRNA FGD5-AS1, miR-129-5p, and BST2 were associated with macrophage polarization and cervical cancer. We observed that FGD5-AS1 directly targeted miR-129-5p to promote M2 macrophage polarization through inducing BST2 in cervical cancer. Our findings potentially identify novel targets to design therapeutic strategies for cervical cancer.

In our current study, FGD5-AS1 expression was overexpressed in cervical cancer cell lines. Decrease of FGD5-AS1 repressed cell proliferation, migration, and invasion and facilitated apoptosis. These findings provide powerful evidence, which supported that FGD5-AS1 may act as a carcinogenic driver in cervical cancer. FGD5-AS1 promotes lung cancer cell proliferation via sponging miR-107 and upregulating FGFRL1 [29]. In addition, FGD5-AS1/miR-5590-3p can facilitate renal cell carcinoma proliferation and metastasis through ERK/AKT [30].

Within the tumor microenvironment, tumor-associated macrophages are the major inflammatory cells [31, 32]. In general, tumor-associated macrophages can exhibit M2 phenotypes and induce tumor progression [33]. Hence, identifying the key factors in M2 macrophage polarization is significant for repressing tumor-associated macrophage-mediated tumor progression. To study the mechanisms of tumor-associated macrophage polarization in cervical cancer, we found that decreased expression of lncRNA FGD5-AS1 inhibited M2 macrophage polarization via modulating miR-129-5p and BST2. We reported that FGD5-AS1 could regulate M2 macrophage polarization in cervical cancer.

lncRNAs can display crucial functions in regulating biological cell processes through acting as “sponges” for microRNAs [34, 35]. To study the downstream genes of FGD5-AS1, we used bioinformatics analysis to predict miR-129-5p as a potential miRNA with complementary binding at the FGD5-AS1 3′UTR. miR-129-5p has been reported to participate in cervical cancer. For example, miR-129-5p inhibits cervical cancer progression by inhibiting ZIC2 via downregulating Hedgehog [36]. LINC01305 is able to repress the development of cervical cancer by regulating miR-129-5p and Sox4 [37]. A close association between FGD5-AS1 and miR-129-5p was verified by RIP and luciferase reporter assay. In our study, we observed that miR-129-5p reduced cervical cancer cell growth, which was induced by FGD5-AS1.

It has been reported that BST2 is aberrantly expressed in cancers. Hence, BST2 is attracting much attention in tumors. Upregulation of BST2 indicates nodal metastasis and a bad prognosis in oral cavity cancer [38]. Overexpression of BST2 is associated with poor survival of esophageal, gastric, or colorectal cancer patients [39]. In our study, BST2 expression was significantly elevated in cervical cancer cells. Loss of BST2 obviously repressed cervical cancer cell proliferation, migration, and invasion while triggering cell apoptosis. Moreover, we found that knockdown of BST2 restrained M2 macrophage polarization. BST2 was predicted and confirmed as a target for miR-129-5p in cervical cancer. We implied the effect of BST2 in M2 macrophage polarization in cervical cancer. In our future study, we will collect enough human cervical cancer tissues to detect FGD5-AS1, miR-129-5p, and BST2 expression.

In summary, our data focused on the association between FGD5-AS1, miR-129-5p, and BST2 and demonstrated that FGD5-AS1 promoted M2 macrophage polarization through regulating miR-129-5p-mediated regulation of BST2 in cervical cancer. We suggested that lncRNA FGD5-AS1 could serve as a valuable prognostic indicator for cervical cancer.

## Figures and Tables

**Table 1:** Primers for real-time PCR.

Genes	Forward (5′-3′)	Reverse (5′-3′)
GAPDH	AACGGATTTGGTCGTATTG	GGAAGATGGTGATGGGATT
FGD5-AS1	AGAAGCGGAGGGGTGAAA AT	CCGCCTTATAGTTGGCCCTC
miR-129-5p	ACCCAGTGCGATTTGTCA	ACTGTACTGGAAGATGGACC
BST2	TGTCGCAATGTCACCCATCT	CTTCTCAGTCGCTCCACCTC
CD80	ACGTCAAAGCAGTAGTCAAGG	GGAGGCCCTATGGAAAGTTAC
CD206	ATCCACTCTATCCACCTTCA	TGCTTGTTCATATCTGTCTTCA
CD163	CAGGCATCATCCGCTAT	GGTCTCCGTACCCTCAAT
CD86	ACTGGTGAAGCCAATAACGCA	TCCGTGATGACAACTAGGATCTT

## Data Availability

The data used to support the findings of this study are included within the article.
